# The effects of different intermittent fasting regimens in people with type 2 diabetes: a network meta-analysis

**DOI:** 10.3389/fnut.2024.1325894

**Published:** 2024-01-25

**Authors:** Wen Xiaoyu, Xiao Yuxin, Lai Li

**Affiliations:** School of Nursing, Chengdu Medical College, Chengdu, China

**Keywords:** intermittent fasting, fasting, type 2 diabetes, network meta-analysis, randomized controlled trials

## Abstract

**Objective:**

To compare the effects of four intermittent fasting regimens on blood glucose and insulin sensitivity in people with type 2 diabetes.

**Methods:**

Randomized controlled trials of intermittent fasting in the treatment of patients with type 2 diabetes mellitus in PubMed, the Cochrane Library, Embase, ScienceDirect, Web of Science, CNKI, VIP Database, and WANFANG Database were searched for from the library to September 2023. 2 review authors independently screened studies and extracted data. RevMan 5.4 was used for direct comparison of meta-results. Network meta-analysis was performed using Stata16 software.

**Results:**

13 studies with a total of 867 patients were included. The intervention effects of twice-per-week fasting, fasting-mimicking diet, time-restricted eating, and peridic fasting were better than that of conventional diet. The results of the network comparison showed that there was no significant difference in the intervention effect of the intermittent fasting regimens. SUCRA ranking results showed that the twice-per-week fasting was best for comprehensive interventions for improvement.

**Conclusion:**

From the perspective of fasting blood glucose, glycated hemoglobin and insulin resistance, the twice-per-week fasting intervention has a good effect, which can be used as a reference for patients with inter-type 2 diabetes to choose intermittent fasting regimen. However, more clinical trials are needed to verify this at a later stage.

## 1 Background

Intermittent fasting (IF), an emerging modified fasting programme, differs from the traditional calorie restriction (CR). Compared to continuous CR, IF restricts food intake for a period of time but does not change the type or amount of food consumed for the rest of the time, making it easier to adhere to the benefits of fasting in the long run (1, 2). Three common IF regimens are available (3, 4): time-restricted eating (TRE), alternate-day fasting (ADF), and the twice-per-week fasting (TWF).

Time-restricted eating (5) is the practice of limiting your eating to a specific amount of time per day (usually 4 to 10 h), and the rest of the time you can consume calories according to your own habits.

Alternate-day fasting is when eating days alternate with fasting days (6–8), and on fasting days participants can choose to drink only water, which is called zero-calorie ADF.

Twice-per-week fasting is a modified version of ADF (6) and refers to 2 days of fasting per week, including 2 consecutive or non-consecutive days of fasting per week. In the common protocol of 2-day fasting (7), there is an option to consume 0–25% of calories.

In addition, periodic fasting (PF) and fasting-mimicking diets (FMD) have also been considered as new types of dietary interventions involved in dietary research in type 2 diabetes. PF is a prolonged period of severe calorie restriction or water-only fasting, ranging in duration from 48 h to 1 week, usually every 2 weeks, and in most cases limited in the number of times a year a PF can be carried out. FMD (provided by L-Nutra, Los Angeles, California, USA) is a specially formulated, calorie-restricted, 5-day nutritional program (9, 10).

Dietary intervention is a commonly used therapeutic measure for patients with type 2 diabetes mellitus (T2DM), and it is also the simplest, most effective, and most cost-effective intervention. Traditional dietary interventions achieve glycemic control by restricting caloric restriction by limiting the patient’s total daily intake (11). Appropriate restriction of caloric intake has a positive impact on health (12), but prolonged caloric restriction leads to poor patient compliance (13).

There are more intervention programs for IF, and it is not yet clear which program has the best effect on metabolic improvement in patients with T2DM. Network meta-analysis can indirectly compare different intervention studies on the same study population to derive the best intervention regimen. Therefore, this study compared five IF regimens by network meta-analysis with the aim of providing evidence-based medical evidence for clinical dietary interventions in patients with T2DM.

## 2 Methods

### 2.1 Searches

A computerized search was conducted for Chinese and English Randomized controlled trials (RCTs) published from the time of library construction to September 25, 2023, on the effects of intermittent fasting on interventions for T2DM patients. A total of 8 databases were searched. Chinese databases included China National Knowledge Infrastructure (CNKI), VIP databases, and WANFANG databases; English databases included PubMed, the Cochrane Library, Embase, ScienceDirect, and Web of Science. Relevant systematic evaluations and references to the included literature were traced to supplement unretrieved studies. Searches were conducted by combining medical subject terms and paragraph terms and adapting to database characteristics. Chinese search terms included “intermittent fasting,” “fasting,” and “type 2 diabetes.” English search terms included “intermittent fasting,” “fasting,” “non-insulin-dependent diabetes,” “T2DM,” “type 2 diabetes,” and “randomized controlled trials.”

### 2.2 Selection criteria

The study inclusion criteria strictly followed the PICO principles and included intervention studies where the study methodology was a RCT and the language was English or Chinese. The requirements for inclusion and exclusion criteria were as follows:

The inclusion criteria were as follows: (a) Participants: Type 2 diabetics (age > 18 years, meeting the diagnostic criteria for diabetes mellitus published by the World Health Organization in 1999); (b) Interventions: Interventions include TWF, TRE, or intermittent fasting such as PF, FMD, and other intermittent fasting regimens; (c) Comparisons: The control group intervention was either a basal diet or a diabetic diet; (d) Outcomes: glycated hemoglobin (HbA1c), fasting glucose (FPG), insulin resistance (HOMA-IR).

We excluded studies that: (a) duplicate published studies; (b) study protocols, summary of the meeting, and case reports; (c) missing or misreported outcome indicators; (d) no literature reporting the outcome indicators in this study; (e) different timing of interventions in the trial and control groups.

### 2.3 Screening procedure

After deleting duplicates and manually checking for duplicates through Endnote X9 software, literature screening and data extraction were completed independently by two investigators trained in evidence-based methods based on selection criteria to select studies that meet the inclusion criteria.

If there were disagreements during the article screening process, they were resolved through discussion between the two individuals or with the assistance of a third investigator. The researchers selected qualified personnel who had received evidence-based training in the school curriculum and participated in evidence-based competitions. Literature screening was done through a 2-step process of reading the title and abstract and reading the full text. An Excel sheet was used to extract literature information, including author, year, type of study, sample size of study population, intervention, and outcome indicators.

### 2.4 Quality assessment

The quality assessment of the included studies was performed using the PRISMA statement (14), as detailed in the supplementary material. The risk of bias was assessed using the evaluation criteria for randomized controlled trials in Cochrane Handbook 5.1.0 (15). The risk of bias in the included studies was evaluated for veracity in seven aspects, including randomization method, blinding, allocation concealment, selective reporting, completeness of outcome data, and other biases. Evaluators were required to make a judgment of low risk of bias, high risk of bias, and unclear for each item.

If the study fully met these criteria, there was a low likelihood of all kinds of bias, and the quality grade was A. If these criteria were partially met, there was a moderate likelihood of bias, and the quality grade was B. If these criteria were not met at all, there was a high likelihood of bias, and the quality grade was C. And then it was cross-checked, and if there were any disagreements, they were resolved through discussion or referred to a third researcher for discussion and resolution. The final quality evaluation resulted in the inclusion of studies with quality grades A and B in this study.

### 2.5 Data analyses

RevMan 5.4 was used for direct comparison, and I^2^ > 50% and *p* < 0.05 were considered to be heterogeneous; Stata 16 software was used for reticulation meta-analysis. Firstly, a consistency test was carried out; if the evidence network did not exist in a closed loop, then the consistency model was used for analysis; if there was a closed loop, then the node splitting method was used to carry out a local inconsistency test; and if the results prompted significant inconsistency, then the consistency model was used for reticulated meta-analysis; otherwise, the inconsistency model was chosen to carry out the analysis.

The area under the curve (SUCRA) of the cumulative probability plot was used to rank the individual outcome indicators, and the larger the area, the better the intervention effect and the greater the likelihood of being the best evidence for the intervention. Comparison-correction funnel plots were used to evaluate whether there was a small-sample effect or publication bias for the interventions.

## 3 Results

### 3.1 Study characteristics

**Figure 1:**
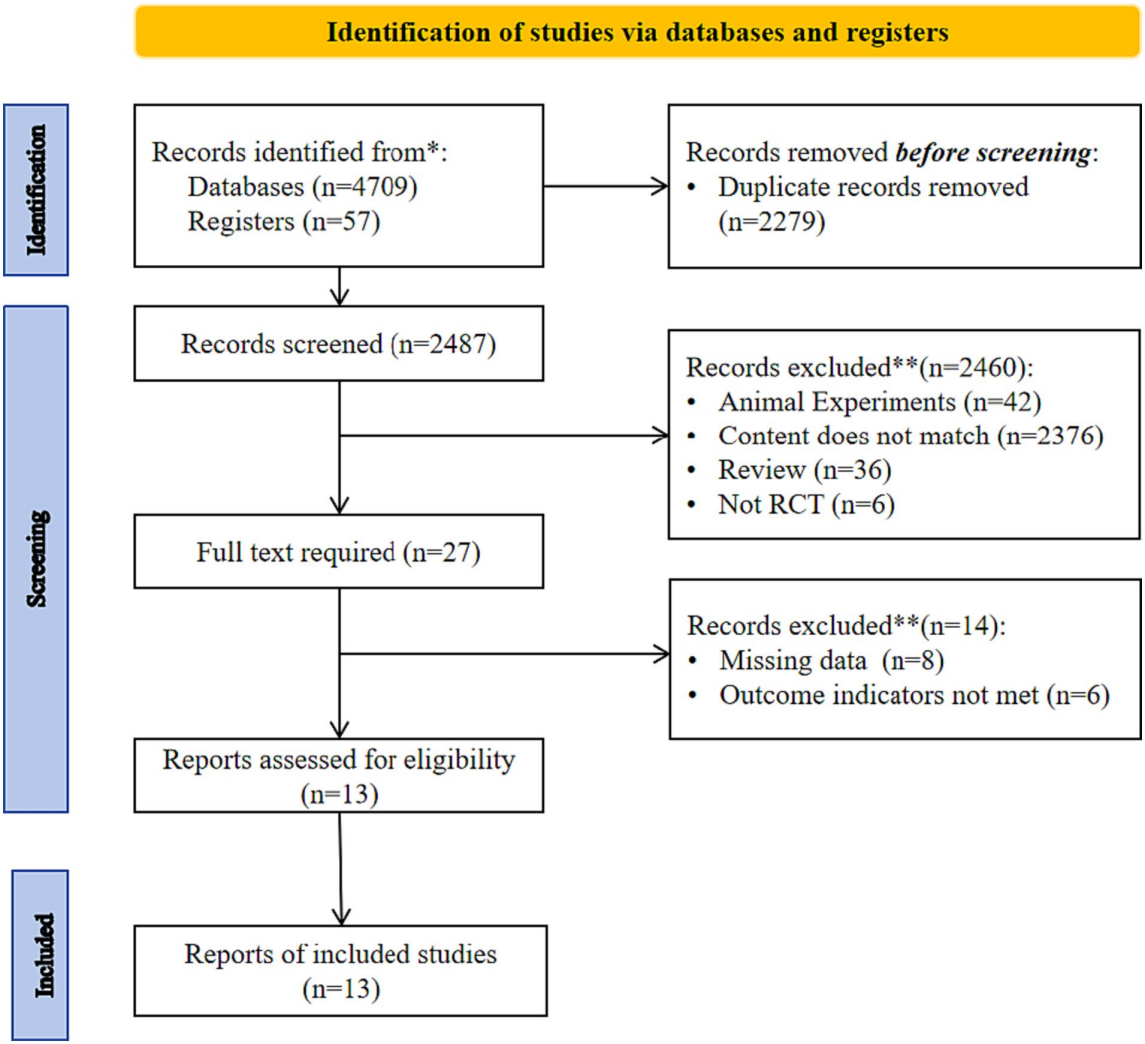
Flowchart of article selection for meta-analysis.

Figure 1 shows the study screening process. After removing duplicates, 2,487 articles were initially included, and after reading the titles, abstracts, and full text, 13 studies (16–28) were finally included.

Table 1 shows the basic characteristics of the included studies. Of the 13 included RCTs, 10 were in English (16, 17, 20–26, 28) and 3 were in Chinese (18, 19, 27), for a total of 867 patients with T2DM.

**Table 1:** Characteristics of included studies.

Author, year	Number		Interventions		Outcomes
	Experimental	Control	Experimental	Control	
(21)	22	24	TRE	regular diet	①
(28)	32	31	FMD	regular diet	②
(24)	7	7	Early TRE	calorie restriction	②
(25)	14	12	TWF	regular diet	①②③
(18)	39	38	TRE	regular diet	①②③
(17)	54	50	TRE	regular diet	①②③
(23)	50	50	FMD	regular diet	①②
(27)	32	33	PF	regular diet	①②
(19)	30	30	FMD	regular diet	①②③
(16)	70	67	TWF	calorie restriction	①②
(20)	16	16	FMD	regular diet	①③
(22)	30	29	FMD	regular diet	①②
(26)	16	15	FMD	regular diet	①②

①glycated hemoglobin; ②fasting glucose; ③insulin resistance.

**Figure 2:**
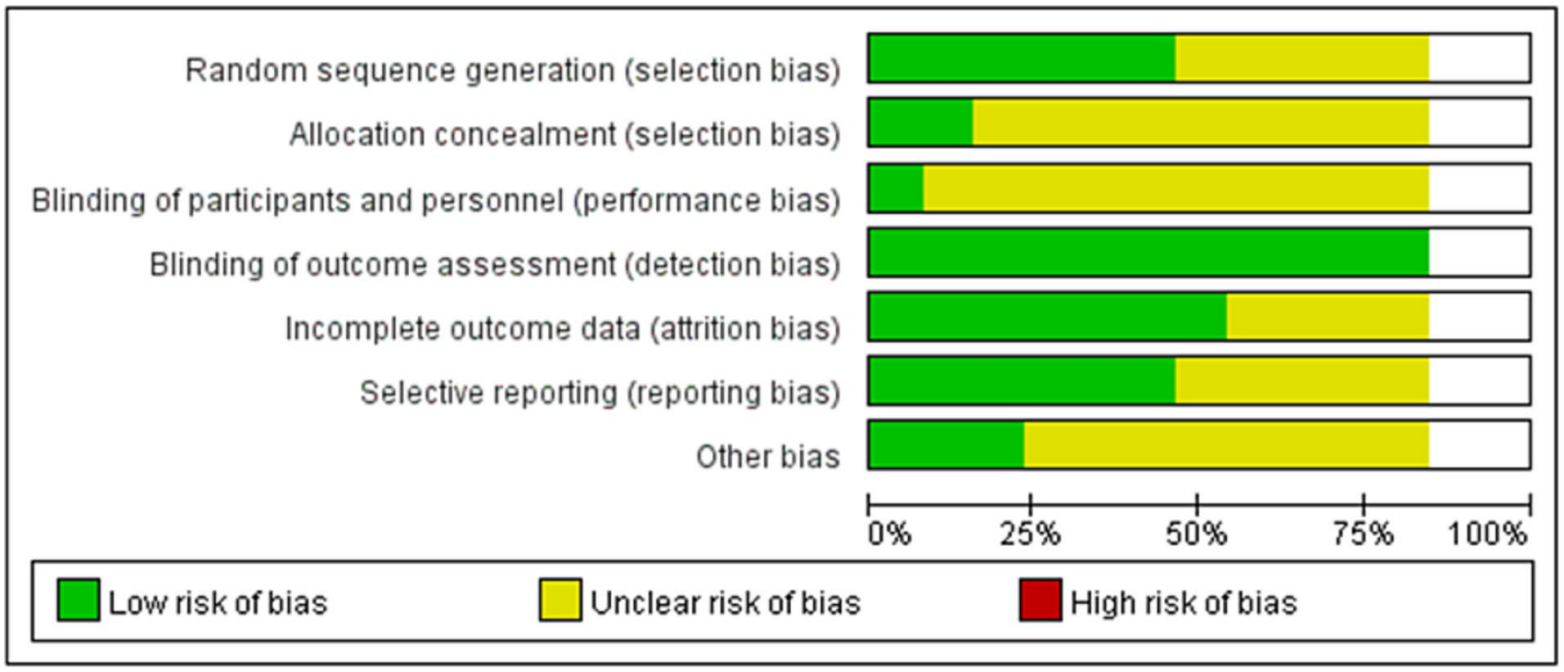
Risk of bias assessment.

The risk of bias in the included studies is shown in Figure 2. The quality of the 13 studies was B. 6 of the studies were allocated according to the randomized numeric table method (17, 19, 20, 23, 25, 28), and the rest mentioned randomization only; 2 studies mentioned allocation concealment (17, 20); and all studies explained the patients’ loss of visits and the reasons for it.

### 3.2 The reticulation of different interventions and its consistency test

**Figure 3:**
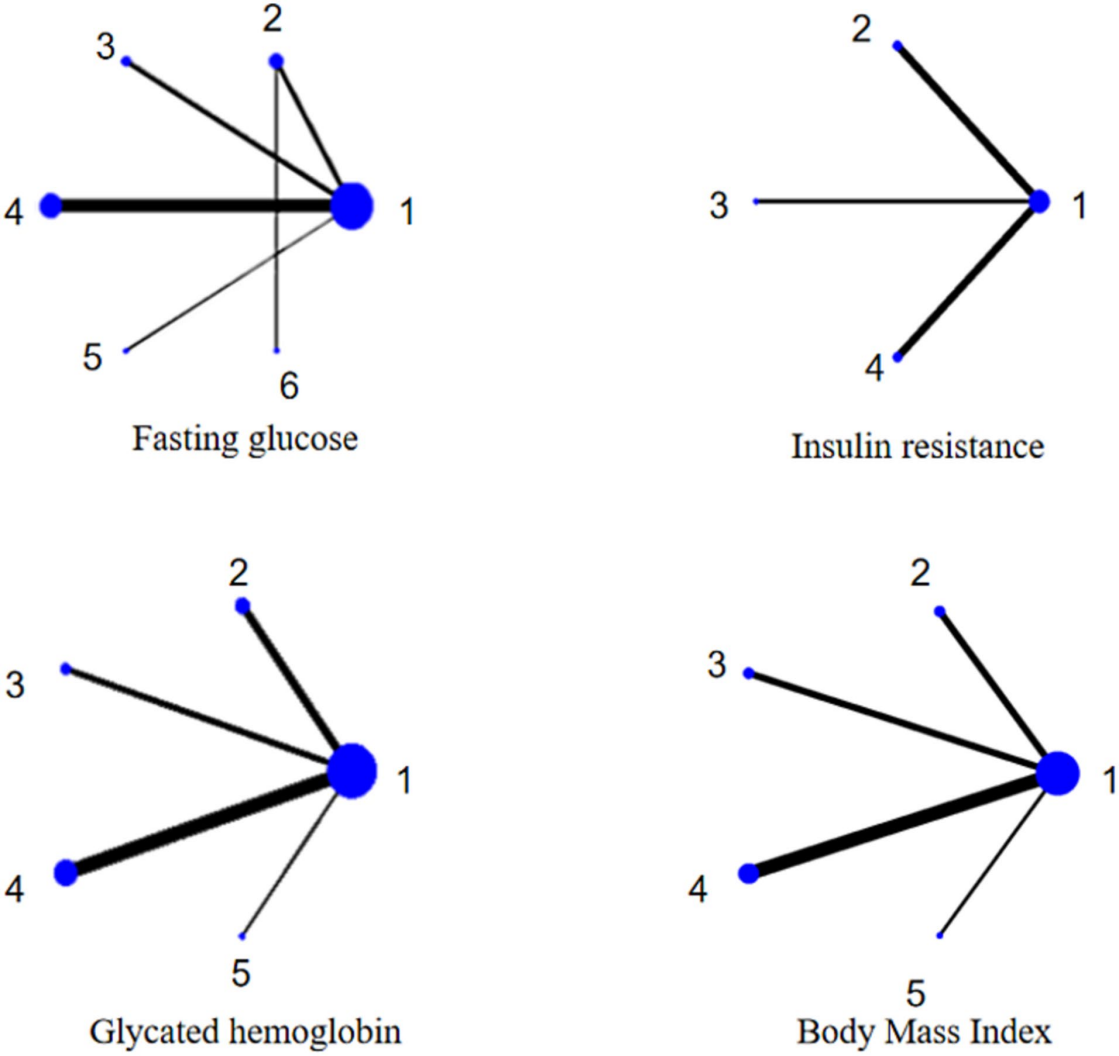
Evidence network. 1 regular diet; 2 TRE; 3 TWF; 4 FMD; 5 PF; 6 calorie restriction.

Figure 3 shows that there are no closed loops in the web of relationships between the different interventions and that no inconsistency tests are needed.

### 3.3 Insulin resistance

A total of 5 RCTs were performed (2, 9, 13, 15, 31). Conventional meta results showed a statistically significant difference (*p* < 0.05) in the combined effect sizes (I2 > 50%) using a random effects model, suggesting that TWF, FMD, and TRE were superior to the regular diet.

Table 2 shows the results of the network meta-analysis, with no statistically significant difference between the 3 IF regimens in a two-by-two comparison. Table 3 shows the SUCRA ordering of the effectiveness of the 3 IF interventions on insulin resistance in patients with T2DM: the order of superiority of the results of the 3 IF regimens was:TWF (50.7%) > TRE (42.9%) > FMD (30.7%).

**Table 2:** Results of reticulated meta-analysis of HOMA-IR [*SMD(95%CI)*].

Interventions	TRE	TWF
FMD	−1.23(−10.56,8.09)	−1.88(−13.31,9.55)
TWF	0.65(−10.76,12.06)	-

**Table 3:** SUCRA sorting results.

Outcome	1	2	3	4	5
HOMA-IR	TWF(50.7%)	TRE (42.9%)	FMD (30.7%)	—	—
HbA1c	PF (49.0%)	TWF(47.6%)	TRE (35.0%)	FMD (30.2%)	—
FPG	calorie restriction(74.3%)	TWF(57.3%)	TRE (47.6%)	FMD (36.9%)	PF (8.9%)
BMI	TWF(61.7%)	PF (56.4%)	TRE (55.4%)	FMD (10.9%)	—

### 3.4 Fasting glucose

A total of 11 RCTs were conducted (16–19, 22–28). Conventional meta-analysis used a random effects model to combine the effect sizes (I2 > 50%), and the difference was statistically significant (p < 0.05), suggesting that TWF, FMD, TRE, and PF were superior to the regular diet, and thatTWF was superior to the calorie-restricted diet.

Table 4 shows the results of network meta-analysis, and there was no statistically significant difference in two-by-two comparisons of the 5 IF regimens. Table 3 shows the SUCRA ordering: the order of superiority of the results of the 5 IF regimens was caloric restriction (74.3%) > TWF (57.3%) > TRE (47.6%) > FMD (36.9%) > PF (8.9%).

**Table 4:** Results of reticulated meta-analysis of FPG [*SMD(95%CI)*].

Interventions	TRE	TWF	FMD	PF
Calorie restriction	1.38 (−2.23,5.00)	1.04 (−3.96,6.03)	1.77 (−2.85,6.39)	4.00 (−1.58,9.59)
PF	−2.62 (−6.88, 1.64)	−2.97 (−7.23, 1.30)	−2.23 (−6.05, 1.59)	—
FMD	−0.39 (− 3.27,2.50)	−0.73 (−3.63, 2.16)	—	—
TWF	0.35 (− 3.11,3.80)	—	—	—

### 3.5 Glycated hemoglobin

A total of 11 RCTs were performed (16–23, 25–27). Conventional meta-analysis used a random effects model to combine the effect sizes (I^2^ > 50%), and the difference was statistically significant (*p* < 0.05), suggesting that TWF, FMD, TRE, and PF were superior to the regular diet.

Table 5 shows the results of network meta-analysis, and there was no statistically significant difference between the 4 IF regimens in a two-by-two comparison. Table 3 shows the results of SUCRA sequencing as PF (49.0%) > TWF (47.6%) > TRE (35.0%) > FMD (30.2%).

**Table 5:** Results of reticulated meta-analysis of HbA1c [SMD*(95%CI)*].

Interventions	TRE	TWF	FMD
PF	0.59(−4.50, 5.68)	−0.01 (−5.41, 5.40)	0.71 (−4.13, 5.54)
FMD	−0.12 (−3.35, 3.11)	−0.72 (−4.43, 3.00)	—
TWF	0.60 (−3.44, 4.64)	—	—

### 3.6 Body mass index

A total of 9 RCTs were performed (16–20, 22, 23, 25, 27). Conventional meta-analysis used a random effects model to combine the effect sizes (I2 > 50%), and the difference was statistically significant (*p* < 0.05), suggesting that TWF, FMD, TRE, and PF were superior to the regular diet.

Table 6 shows the results of network meta-analysis, and there was no statistically significant difference between the 4 IF regimens in a two-by-two comparison. Table 3 shows the results of SUCRA sequencing as TWF (61.7%) > PF (56.4%) > TRE (55.4%) > FMD (10.9%).

**Table 6:** Results of reticulated meta-analysis of BMI [SMD*(95%CI)*].

Interventions	TRE	TWF	FMD
PF	0.25(−21.31, 21.80)	−1.07(−22.63, 20.48)	8.76(−10.95, 28.47)
FMD	−8.51(−23.79, 6.77)	−9.84(−25.12, 5.45)	—
TWF	1.32(−16.28, 18.92)	—	—

### 3.7 Publication bias

**Figure 4:**
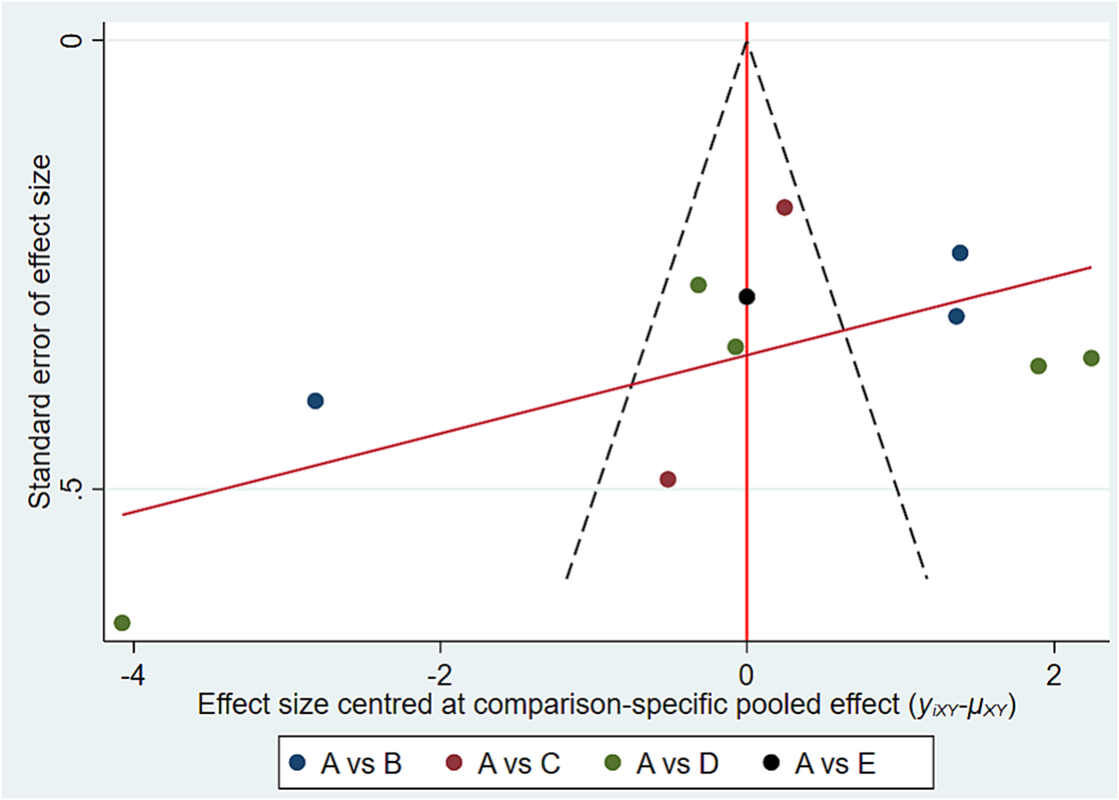
Funnel plot. A (regular diet); B (TRE); C (TWF); D (FMD); E (PF).

Figure 4 shows that a corrected-comparison funnel plot for glycated hemoglobin, an outcome indicator, was plotted to test for publication bias, and the results showed that the scatter of the study was poorly symmetric on both sides of the funnel plot, suggesting that there may be a certain degree of publication bias and small-sample-size potency in this study.

## 4 Discussions

### 4.1 Current status of IF interventions for patients with T2DM

Patients undergoing IF may be better adapted and have improved metabolic markers independently of weight loss compared to traditional continuous energy-restricted dietary intervention programs (29). Studies have shown that IF induces the expression of the histiocyte marker neuronal protein 3 (Ngn3) (30), which leads to the production of pancreatic β-cells and the improvement of pancreatic islet function, and that during fasting interventions, there is an interplay between the feeding/fasting cycle and the autonomic circadian rhythms, which can lead to the normalization of the body’s functioning and the lowering of blood glucose (31).

IF has been proven in terms of safety (32). Subjects experience mild dizziness and constipation in the earliest stages of fasting, but these side effects usually subside by the second week of fasting (1). In addition, dehydration due to insufficient water intake may also contribute to the development of headaches in people with T2DM (32), and ensuring that they drink 1.5 liters of water per day may help alleviate their headaches (33) during the fast.

For most people, the initial feeling of hunger and irritability usually disappears after two weeks to a month as the body and brain adjust to the new habit (34). Therefore, patients are advised to gradually increase the duration and frequency of fasting over a period of several months and should consult a dietitian about the applicability and limitations of their use of dietary interventions before proceeding with IF treatment in order to tailor the optimal IF intervention for each individual.

Among the studies we included, adverse effects were only mentioned in the study by Huang Weixuan et al. This may be due to impaired mitochondrial fatty acid oxidative energy supply as a result of the reduction of free L-carnitine during fasting, and it was hypothesized that the addition of L-carnitine analogues during fasting may reduce the incidence of adverse effects (35, 36).

Currently, IF intervention studies conducted in patients with T2DM have shown some positive effects, but it has not been clarified whether there are differences in the intervention effects of different IF programs. In this study, we used Network meta-analysis to compare the effects of 5 IF regimens on the improvement of glycemia and insulin resistance in patients with T2DM and to explore the optimal IF regimen.

### 4.2 Best effect of combined intervention with TWF

The SUCRA results showed that patients with T2DM on TWF had the best combined effect in improving fasting glucose and insulin resistance compared to other IF regimens, and therefore it has the greatest potential to be the optimal IF regimen. In addition, TWF has demonstrated weight control benefits in previous studies (37, 38), improving adherence compared to daily calorie restriction without compromising weight loss. TWF requires people to follow a very low-calorie diet for only two days of the week, and the rest of the week they do not have to adhere to a strict restriction of calorie intake, which is a significant improvement over other restrictive dietary behaviors (e.g., the ketogenic, vegan, or daily calorie restriction diets). Diets, or daily calorie restriction, are more adaptable than other restrictive dietary behaviors.

Previous clinical trials have been devoted to improving insulin sensitivity to ameliorate the reduced response to insulin in patients with T2DM due to defective insulin secretion from their own pancreatic β-cells or insulin sensitivity. There is now increasing evidence confirming the beneficial effects of IF in animals (39) and T2DM patients (40). The present results show that TWF has a better overall effect on improving insulin sensitivity and therefore can be a preferred option for dietary intervention in patients with T2DM.

### 4.3 TRE interventions are highly effective

SUCRA results show that in addition to TWF, TRE also significantly improves glycemia and insulin resistance in patients with T2DM. TRE divides the day into periods of fasting and periods of eating, and the length of fasting is usually 14–16 h. The 16:8 regimen is more commonly used in TRE regimens, i.e., 8 h of eating and 16 h of fasting. Recently, the 15:9 fasting regimen has also appeared in studies, controlling energy intake all within 15 h. Previous studies have shown that TRE has also shown better results in improving insulin sensitivity (41), and that an increase in fasting duration improves fasting glucose values in patients with T2DM (3). Therefore, clinicians or dietitians can help patients with T2DM understand and co-develop an appropriate IF program.

## 5 Conclusion

This study showed that TWF had a better combined effect on improving blood glucose and insulin resistance in patients with T2DM, but most of the differences between IF measures were not significant. Considering the limitations of the study, clinical staff should still formulate the optimal dietary program in accordance with the patients’ actual conditions and dietary habits to improve treatment compliance.

Intermittent fasting therapy, as a new dietary intervention mode, has certain advantages in the treatment of T2DM, especially TWF. The role of long-term dietary intervention in the treatment of chronic diseases should not be ignored. For individual patients, a pattern of fasting that is most easily integrated into their lifestyle should be chosen to reap the benefits of long-term fasting. In future studies, the time window and caloric range should be further refined to provide an effective, non-drug intervention for the clinical treatment of T2DM patients.

### 5.1 Limitations

The limitations of this study are as follows: firstly, the number of randomized controlled trial studies included in the literature is not enough, most of the studies have not yet reported the blinding and allocation concealment scheme, and a certain amount of selective bias is considered to exist. Secondly, the included studies had small sample sizes and lacked proof of large sample sizes, which may reduce the credibility of the findings. Then, the dietary habits of domestic and international study subjects were different, and there were differences in the types of diets, which may affect the results to some extent. Finally, the inconsistency of the content of conventional treatment protocols may cause clinical heterogeneity, but the limitation of the number of studies does not allow further in-depth analysis. In summary, in addition to the conventional dietary regimen, TWF is more effective in improving blood glucose and insulin resistance in patients with T2DM, and there is no occurrence of adverse reactions for the time being.

## Data availability statement

The original contributions presented in the study are included in the article/supplementary materials, further inquiries can be directed to the corresponding author.

## Author contributions

XY: Data curation, Software, Writing – original draft, Writing – review & editing. WX: Data curation, Investigation, Methodology, Writing – original draft, Writing – review & editing. LL: Supervision, Writing – review & editing.
